# COVID-19 Among Omani Pilgrim Returnees From the Ashura Mass Gathering

**DOI:** 10.7759/cureus.36003

**Published:** 2023-03-10

**Authors:** Tawfiq Al Lawati, Zahra T Al Lawati, Bassim Al Bahrani, Salah Al Awaidy

**Affiliations:** 1 Pediatrics, The Royal Hospital, Muscat, OMN; 2 Computer Sciences, Freelance, Muscat, OMN; 3 National Oncology Center, The Royal Hospital, Muscat, OMN; 4 Epidemiology and Public Health, Oman Ministry of Health, Muscat, OMN

**Keywords:** iraq, covid-19 prevention, covid-19 vaccination, hand wash, face mask, covid-19, sars-cov-2, mass gatherings, pilgrims

## Abstract

Background

The annual Ashura pilgrimage is a mass Islamic gathering during which millions of worshippers converge in the city of Karbala in Iraq. We report on the incidence of the coronavirus disease 2019 (COVID-19) in Omani pilgrims returning from Karbala in the month of Muharram (August) 2021 during the COVID-19 pandemic.

Methodology

This is a retrospective study using an electronic, self-completed, and Arab-language survey, composed of 17 questions, that was distributed to all pilgrims returning from Karbala. Participation was voluntary, and consent with confidentiality was obtained. Data on the demographics including sex, COVID-19 vaccination record, type of vaccine, duration of stay, compliance with wearing a face mask, using hand sanitization, and polymerase chain reaction (PCR) results for severe acute respiratory syndrome coronavirus 2 (SARS-CoV-2) virus before the departure from Oman, upon the return to Oman, and on the eighth post-quarantine day were collected. The responses were collected from the period between August 28, 2021, and September 25, 2022.

Statistical association and analysis were performed using the Statistical Package for Social Sciences (SPSS) software (IBM SPSS Statistics, Armonk, NY).

Results

Out of 250 pilgrims, 139 responded to the survey. Fifty-two participants (37.4%) were males, and 87 (63.6%) were females. None of the pilgrims had positive SARS-CoV-2 PCR results before their departure from Oman. Only four pilgrims (2.9%) were detected positive on PCR by the end of a compulsory quarantine on the eighth day after arrival to Oman. No hospital admissions were recorded. The vast majority of the pilgrims were vaccinated with two doses of COVID-19 vaccination, while some few pilgrims were not vaccinated at all. Most of the pilgrims were also compliant with mask wearing, and just over half the pilgrims were compliant with hand sanitization. No significant statistical association was found between contracting SARS-CoV-2 virus infection and taking SARS-CoV-2 vaccination, the number of vaccination doses, having had COVID-19 before, wearing a mask, or compliance with hand sanitization.

Conclusion

The incidence of COVID-19 cases among pilgrims returning from Iraq during the COVID-19 pandemic was low. No significant difference was noted between pilgrims vaccinated and compliant with the protective measures and those who were not vaccinated or compliant. Herd immunity could be a possible explanation for the low incidence of COVID-19 infection. Larger studies are needed to investigate the incidence of COVID-19 in Ashura pilgrims.

## Introduction

Severe acute respiratory syndrome coronavirus 2 (SARS-CoV-2) infection is caused by the coronavirus and leads to severe lung infection in humans [1]. The pandemic was proclaimed on March 11, 2020, by the director-general of the World Health Organization [2]. Religious congregations have been associated with the spread of the SARS-CoV-2 virus and other respiratory viral infections in many parts of the world because of the congestion. Examples of such gatherings include the Tabligh Muslim gathering held in February 2020 in Malaysia resulting in over 1,500 cases of coronavirus disease 2019 (COVID-19) [3]. In a recent study by Al-Ansari et al. on the pilgrims of Al-Arbaeen Walk toward Karbala, Iraq, in October 2018, 1,842 pilgrims were interviewed regarding respiratory symptoms and the precautions taken to prevent the spread of respiratory infection. The pilgrims were asked in a direct interview about hand washing, mask wearing, and having fever or any respiratory symptoms. The study showed that about 87% of the participants developed a cough and 95% developed a fever both after joining the walk [4]. The study indicated a rapid spread of respiratory symptoms among the pilgrims even being in an open walking area. The spread of respiratory viral infections has also been reported during Al-Haj season in Makkah by Memish et al., who reported an increase in respiratory viral infections from about 7% before Haj to 45% after Haj in a sample of 1,200 pilgrims in the year 2013 [5]. Therefore, Saudi Arabia’s health authorities limited the number of Haj pilgrims for 2020 and 2021 to only less than a million [6] for fear of a massive outbreak of COVID-19. Zero cases of COVID-19 were reported by the Saudi authorities during the Haj season in the year 2021 [7].

Annually, and during the month of Muharram and similar to the Arbaeen season, millions of Muslim pilgrims converge to the holy city of Karbala, commemorating Ashura and making it one of the largest annual mass gatherings in the world.

August 2021 coincided with the month of Muharram. Six million people including Omani pilgrims visited Karbala [8] when COVID-19 was rampant in Iraq, with a daily caseload of around 7,000-8,000 and 60-70 deaths [9]. By August 2021, SARS-CoV-2 virus infected 201,516,060 people and claimed 4,276,254 lives worldwide (case fatality ratio {CFR} of 2.1%) [10].

The travel regulations in Oman mandated two doses of COVID-19 vaccination and one negative polymerase chain reaction (PCR) for the SARS-CoV-2 virus prior to the travel to Iraq. In addition, a negative SARS-CoV-2 PCR was required to board the flight back to Oman and a repeat PCR test at the Omani airport upon arrival. A final PCR test was required from all the pilgrims at the end of a compulsory self-quarantine period of eight days. All pilgrims were instructed to comply with hand hygiene and wear a face mask while visiting the holy places in Iraq. In this study, we report on the incidence of COVID-19 in Omani pilgrims returning from Karbala, Iraq, during the month of Muharram (August) 2021.

## Materials and methods

Study design and participants

Three groups of Omani citizens headed to Karbala in 2021. Group A had 20 pilgrims, group B had 105 pilgrims, and group C had 125 pilgrims. All groups had a leader who coordinated their travel requirements and was in charge of their well-being. Upon return from Karbala, all group leaders were contacted by phone by the investigators and invited to participate in the study. The pilgrims, in turn, were invited by their group leader to participate voluntarily in the study. The study was limited to the pilgrims to Karbala.

The group leaders were asked to distribute through the WhatsApp application an Arabic language, self-elaborated, 17-question survey using Google Forms (Google, Inc., Mountain View, CA). The survey contained an introduction to the study, an invitation to voluntarily participate, and the principal investigator’s (PI) phone number. Appendix 1 illustrates all questions in the survey.

Filling out the electronic form was considered consent to participate in the study. Written consent was not feasible as pilgrims lived as far as 300 km from the PI. The survey was first piloted on five people to assess the acceptability and understanding of the pilgrims to the forms. All the comments and corrections were adopted prior to sending the survey to the group leaders of the pilgrims.

The survey questions enquired about the age, sex, prior infection with COVID-19, duration of stay in Iraq, type and doses of vaccination, compliance with hand sanitization, frequency of wearing a face mask, face mask type, and the PCR report of SARS-CoV-2 upon arrival to Oman and on the eighth post-quarantine day.

Pilgrims were considered compliant with mask wearing if they wore the mask always or most of the time. Noncompliance was considered if the pilgrim wore the mask occasionally or rarely. Wearing a face mask and hand sanitization were considered non-pharmaceutical, while vaccination was referred to as a pharmaceutical measure of prevention of COVID-19.

Pilgrims who were not reached through the WhatsApp application were contacted by phone by the investigators and were requested to fill out the form. Those who were not familiar with Google Forms were advised to get help from a family member. The pilgrims returned to Oman in mid-August 2021, and all data were collected electronically through Google from August 28, 2021, to September 25, 2022.

Sample size

A total of 250 Omani pilgrims were identified from the records of the leaders of the travel groups to have visited Iraq during the Ashura season in August 2021. A sample size of 152 respondents was required to ensure a confidence level of 95%, a rate of response of 50%, and a margin of error of 5%, as calculated using Raosoft [11].

Statistical analysis

Continuous variables were reported as mean with standard deviation or median with interquartile range. The categorical variables were described as numbers and percentages. Chi-square tests or Fisher exact test was used for categorical variables. A p-value of less than 0.05 was considered statistically significant. Statistical analyses were performed with Statistical Package for Social Sciences (SPSS) (version 20.0) (IBM SPSS Statistics, Armonk, NY).

Ethics and informed consent

The survey form included an introductory statement on the voluntary and confidential nature of the study, and the phone number of the principal investigator was put in for any queries. Formal ethical approval was granted from the Health Studies and Research Approval Committee (HSRAC) of the Ministry of Health of Oman (approval number: MoH/DGPS/CSR/HSRAC/1/2023).

## Results

The survey was sent out to 250 pilgrims and 139 pilgrims. One hundred and thirty-nine (56%) pilgrims responded to the survey, leading to a confidence level of 90%. There were 52 (37.4%) males and 87 (63.6%) females. The mean age of the pilgrims was 42.6±13.7 years, and the median was 42.0 years (range: 15-76 years). Table 1 shows the demographic data of the group with the length of stay in Iraq and prior acquisition of COVID-19 by the pilgrims.

**Table 1:** The demographic characteristics of pilgrims with the duration of stay

Demographic Feature	Frequency	Percentage
Sex		
Male	52	37.4
Female	87	62.6
Mean age	42.6±13.7	
Age categories (years)		
<18	3	2.2
18-40	63	45.3
41-60	60	43.1
>60	13	9.4
Duration of stay (days)		
≤10 days	102	73.4
>10 days	37	26.6

Sixty (43.2%) pilgrims had COVID-19 before their travel with a mean time of 2.5±1.4 months. Upon returning from Iraq at Oman International Airport, only two pilgrims (1%) had a positive PCR test for SARS-CoV-2. Their PCR positivity remained through to the eighth day of the institutional quarantine. On day 8 and while under institutional quarantine, two more pilgrims turned positive for SARS-CoV-2 on a PCR test, raising to a total of four people and an incidence of 2.9%. Pilgrims with COVID-19 were asymptomatic, and none was hospitalized by the second week of arrival.

Out of the total, 132 (95%) pilgrims received at least a single dose of vaccination, 121 (87.1%) received two doses, 11 (7.9%) received a single dose, and seven (5%) were not immunized. Table 2 shows the types of vaccines taken by the pilgrims.

**Table 2:** Compliance with immunization and non-pharmacological protective measures against COVID-19 COVID-19: coronavirus disease 2019

Protective Measure	Frequency	Percentage
Doses of vaccination		
2 doses	121	87.1
1 dose	11	7.9
Not vaccinated	7	5.0
Time of the second dose of vaccination prior to travel		
<30 days	23	16.5
30-60 days	46	33.1
>60 days	55	39.6
Type of vaccination		
Pfizer	109	78.4
AstraZeneca	18	12.9
Others	12	8.6
Frequency of mask wearing		
Always	72	51.8
Most of the time	39	28.1
Rarely	15	10.8
Sometimes	13	9.4
Type of mask		
N95	17	12.2
The common surgical mask	107	77.0
Others	15	10.8
Hand sanitization		
Yes	82	59.0
No	57	41.0

Out of the four positive pilgrims who tested positive for SARS-CoV-2 PCR at the end of the quarantine period, three were vaccinated with two doses, and one was unvaccinated. Out of the seven pilgrims who were not vaccinated, one had no previous COVID-19, and one was not sure of a previous infection.

Seventy-two (52%) of the pilgrims reported wearing a face mask always, and 39 (28%) reported wearing a mask most of the time. A total of 111 pilgrims (79.9%) were considered compliant with face mask wearing. On the other hand, 28 pilgrims (20%) were non-compliant and reported not wearing or rarely wearing a face mask. Table 2 demonstrates the types of masks used by the pilgrims.

Only two pilgrims out of the four who were positive by the end of the quarantine period were compliant with wearing a face mask. With regard to hand sanitization, 82 pilgrims (59%) reported using hand sanitizers regularly, while 39 (41%) were not compliant with hand sanitization.

Regarding a previous infection with COVID-19, 60 pilgrims (43.2%) had a previous infection, 72 (52%) reported not being infected, and seven (5%) were not sure about any previous infection. Two out of the four who were positive for SARS-CoV-2 PCR reported being infected earlier with COVID-19 infection.

Table 2 summarizes the frequency of pilgrims with their compliance with the vaccination and the non-pharmacological protective measure against COVID-19.

There was no significant association found between receiving COVID-19 vaccination, duration of stay in Iraq, compliance with face mask wearing, regular hand sanitization, and previous infection with COVID-19 with SARS-CoV-2 positivity on day 8 of quarantine (Table 3).

**Table 3:** Association between the protective measures and PCR SARS-CoV-2 test on day 8 from arrival from Iraq COVID-19, coronavirus disease 2019; SARS-CoV-2, severe acute respiratory syndrome coronavirus 2; PCR, polymerase chain reaction

Protective Measure	Positive on Day 8	Negative on Day 8	P-Value
Vaccine doses			0.721
1 dose	3	118	
2 doses	1	10	
No vaccine	0	7	
Vaccine brand			0.844
Pfizer	4	103	
AstraZeneca	0	18	
Others	0	112	
Timing of the second vaccine			0.271
>30 days	101	72.7	
<30 days	23	16.5	
Had a previous COVID-19 infection			0.567
Yes	2	56	
No	2	70	
Maybe	0	7	
Mask wearing			0.104
Always	1	69	
Most of the time	1	38	
Rarely	0	15	
Sometime	2	11	
Hand sanitization			0.661
Yes	2	78	
No	2	55	

Out of all 139 respondents, only 21 pilgrims were compliant with taking at least one dose of vaccine, hand sanitization, and face mask wearing and also had earlier COVID-19 disease as means of protection of the acquisition of COVID-19 while in Karbala. Out of the 21 pilgrims who had all the protective measures, only one pilgrim (4.8%) tested positive for SARS-CoV-2 PCR on the eighth day of the quarantine. There was no single pilgrim in the group who was not vaccinated and at the same time was not observant to hand sanitization and face mask wearing or had no previous COVID-19 disease.

## Discussion

The present study is the first to investigate the incidence of COVID-19 in pilgrims returning from Ashura religious gatherings. The study demonstrated a strikingly low incidence of COVID-19 infection of 2.9% with no hospitalization among pilgrims returning from Karbala. While the general recommendation from all the governments was not to visit SARS-CoV-2-endemic areas such as Iraq, our population took the risk of travelling to Karbala. The low incidence of COVID-19 in our group was unexpected in the setting of six million pilgrims and the absence of strict governmental policies with regard to the SARS-CoV-2 spread. Ashura pilgrimage was unlike the Haj 2021 season, where pilgrims were limited to only one million with strict observation of the preventive measures. There were zero cases of COVID-19 in Al Haj 2021 season [7].

The response rate of 56% is considered above average, as the average response rate on the online survey is about 44% as reported in a meta-analysis by Wu et al. [12].

Our study did not show any association between wearing a mask and contracting COVID-19. The protective effect of face mask on COVID-19 infection was described by Wang et al. even before the era of vaccination. Wang et al. described 278 healthcare workers (HCWs) who were compliant with face mask wearing in the Wuhan region of China and compared them to 215 HCWs who did not wear masks [13]. None of the face mask-wearing group contracted COVID-19, compared to nearly 5% of the non-compliant group. Furthermore, a meta-analysis by Liang et al. reported that the risk of transmission of COVID-19 dropped by 80% by just wearing a mask [14].

With regard to SARS-CoV-2 vaccination, 72.7% of the pilgrims were adequately immunized with two doses for more than a month prior to the travel, which makes them adequately immune [15]. Our study, however, did not show any statistically significant difference between the vaccinated and unvaccinated individuals with regard to the acquisition of SARS-CoV-2 infection. This is again unlike the world literature where COVID-19 vaccination has shown clear protective effects against COVID-19 [16].

The nonsignificant association between face mask wearing and vaccination with COVID-19 infection might raise the issue of herd immunity of the pilgrims due to the widespread COVID-19 vaccine and infection [17]. In fact, out of the seven pilgrims who were not vaccinated, only one thought that he had had no COVID-19 infection prior to travel, and one was not sure if she/he contracted COVID-19 earlier. It is possible that those who had no immunization and still did not contract COVID-19 infection in Iraq were actually immune from possible earlier COVID-19 infection. Recall bias of having earlier COVID-19 is possible, and hence, it is possible that all those who were not immunized actually have had an earlier infection and developed immunity. Herd immunity is acquired by immunization or a previous infection in the community [18].

The possibility of the immunity of the pilgrims still holds true for the rest of the protective factors including hand sanitization, which also showed no significant association with SARS-CoV-2 infection. Hand hygiene has earlier been shown to confer protection from SARS-CoV-2 infection [19].

On the other hand, the findings of the non-significance of all of the vaccination, face masks, hand sanitization, and previous acquisition of COVID-19 might raise concerns about the validity of the findings. It is possible that the relatively small sample, a small number of unvaccinated pilgrims and those who got COVID-19 infection, did not power up to detect a significant association. Regardless of the statistical association between the protective factors and the actual COVID-19 infection, the unexpectedly low prevalence of only 3% in a large mass gathering in an endemic area remains by itself a finding that is not disputable. Unfortunately, there were no data on the mass screening of asymptomatic people in Oman during the study period to compare our findings with the general Omani population. However, an older study by Al-Abri et al. reported an increased seroprevalence in Omani residents from 5.5% in August 2020 to 22% in November 2020, which was the period of the study. Our figure of 2.9% is certainly lower, and the low infection rate might again be explained by the herd immunity of the pilgrims [20].

The pilgrims who were not vaccinated are of interest not only from the point of investigating their antibodies to COVID-19 but also for exploring the reasons for not having the immunization. The fear of vaccination and the distrust of the manufacturing process were the main reasons for vaccine refusal in a recent study on 444 people from Saudi Arabia [21].

The main study limitation is the smaller size than the desired sample to reach a 95% confidence level. Nevertheless, while the confidence level was not 95%, it remained above 90% with 153 responders. Moreover, the small number of pilgrims who had no vaccination has probably skewed the population and the statistic toward no effect. Also, research on pilgrims who had no vaccination is currently hard to find. Furthermore, the self-reported information by the participants about their symptoms, the results of PCR, and admission to a hospital is a source of potential inaccuracy as COVID-19 is still considered a stigma and people could hide the information.

We also acknowledge that there was no robust validation of the study by having a limited pilot sampling. However, for any concerns of the participants during the study, assistance was offered by the PI through the contact number provided.

It is also essential to note that our study was conducted prior to the propagation of the Omicron variant of the SARS-CoV-2 virus. Our finding, hence, may not be fully applicable to the Omicron variant of SARS-CoV-2 virus infection. Nonetheless, vaccination against SARS-CoV-2 and the non-pharmaceutical interventions remain the same for all variants of the SARS-CoV-2 virus.

## Conclusions

The current study revealed a low incidence of COVID-19 infection among pilgrims returning from the endemic area of Karbala in Iraq. There was no association between immunization, hand washing, and mask wearing with the acquisition of COVID-19 disease. Herd immunity might have played a role in this low incidence of COVID-19. Further larger studies looking at the incidence of COVID-19 at the Ashura mass gathering are required.

## Appendices

Appendix 1

Dear pilgrim,

This is a follow-up study of pilgrims who returned from Karbala. The study looks at their health details regarding SARS-CoV-2 based on their PCR results on the eight day of quarantine as mandated by Sultanate of Oman regulations.

Kindly participate in the study to assess the quarantine’s efficacy, and the study will act as a guide for future visits to Karbala.

The study is based on optional real names, or you wish to use a symbolic representation. The study is voluntary and is only for pilgrims to Karbala for Muharram 1443. Kindly distribute the survey to all concerned with the mentioned trip only.

Investigator: Dr. XXXX; telephone: XXXXX

1)      Your real or symbolic name:

2)      Gender: male or female

3)      Age in years:

4)      Tour operator name:

5)      Duration of visit:

●       <7 days

●       7 days

●       ≤10 days

●       >10 days

6)      Doses of COVID-19 vaccination received:

●       Not immunized

●       One dose

●       Two doses

7)      When did you take the second dose?

●       Did not take it

●       <1 month

●       30-60 days

●       >60 days

8)      Type of vaccine:

●       BNT162b2 vaccine

●       AstraZeneca

●       Others

9)      Did you get a COVID-19 infection earlier?

●       No

●       Yes

●       Maybe

10)     When do you think you got the COVID-19 infection?

●       ≤1 month

●       1-2 months

●       3-4 months

●       >4 months

●       Did not get COVID-19 before

11)     Were you wearing the mask when mixing with others?

●       Always

●       Most of the time

●       Sometimes

●       Rarely

12)     What type of mask did you use?

●       N95

●       Common surgical

●       Others

13)     Were you using hand sanitizers regularly?

●       Yes

●       No

14)     Did you experience any COVID-19 symptoms such as fever, cough, sore throat, or diarrhea?

●       Yes

●       No

15)     Was the COVID-19 PCR at the Omani airport negative upon return?

●       Yes

●       No

16)     Did you experience COVID-19 symptoms while in quarantine?

●       Yes

●       No

17)     Was the PCR on day 8 of quarantine negative?

●       Yes

●       No
